# Doctor and Parson

**Published:** 1910-04-16

**Authors:** 


### Doctor and Parson.

It is now some 20 years since "Robert Elsmere," with its profusion of donnish culture, its feminine execution, and its share of that comment of which Mr. Gladstone was rather incontinent, made its author's name and raised a stir over matters with which we have no concern here. But in spite of the frankness of the book in dealing with religious topics, its tone was not anti-clerical by any manner of means. Indeed, Mrs. Humphry Ward dwelt sympathetically upon the social advance made by rural clergy during the first three-quarters of the nineteenth century; as she epigrammatically put it, Dr. Primrose has now given the go-by to Farmer Flamborough. This is so, and the cloth still retains the respect properly due to it as the mark of a learned and reverend profession. But while Mrs. Ward's book was multiplying its editions, another change began, the results of which by now are fairly evident. What need to recount them in their varying magnitude and importance? We see the periodical announcement: "Another Medical Plum," in newspapers of a political complexion commonly called Radical; cabinet ministers in counsel not with bishops but with official medical advisers; the attention paid, even in the staidest intellectual circles, to the deliverances of Mr. W. H. Mallock; a medical curriculum yearly increasing in scope; considerable tightness in ecclesiastical financial matters; Mr. Stephen Paget appealing to medical men to show the clergy how to put down Christian Science; and many other signs of the times. The century end-change, in fact, if not exactly perhaps that the priest is valued less, is certainly that the physician is valued a good deal more.

It is illustrated well by one little passage in the book spoken of. Robert Elsmere, we are told, had been a rector in Westmorland. Generous, hard working, kindly, enthusiastic, he was a general favourite and an entire success. Now one of the touches composing this picture, one of the details of the study, is as follows. He kept, we are informed, a "huge medicine chest" for the benefit (or otherwise) of his parishioners, and—"causticked all the diphtheritic throats in the place with my own hand. Our parish doctor is an infirm old noodle, and I just had to do it." Anyone can see that nowadays this part of the picture is *rococo*. Leaving serum-therapy—which, fortunately, amiable dilettantists as well as astute quacks are wise enough not to meddle with—out of count as an accident of the particular case, we may be certain that at the present day no clergyman worth his salt would behave thus. Whatever the faults of the more fashionable public schools, where, as a class, the clergy of the Establishment are educated, they generally succeed in inculcating common-sense, to say nothing of the habit of gentlemanly conduct. Both qualities teach the useful lesson of minding one's own business, and avoiding impertinence in its etymological as well as in its popular significance. Doubtless school medical officers and country practitioners have to encounter obstacles arising from the propensity of their patients to patent medicines, but any such snatching at the temporal sceptre on the part of Dr. Primrose's latest successors is practically unknown. The old gentleman himself, remiss though he may have been over keeping Farmer Flamborough at his proper distance, would, it may be suspected, never have attempted it.

